# Neurocognitive disorder in Myotonic dystrophy type 1

**DOI:** 10.1016/j.heliyon.2024.e30875

**Published:** 2024-05-08

**Authors:** Stefan Winblad, Olöf Eliasdottir, Sara Nordström, Christopher Lindberg

**Affiliations:** a Icon Lab, Department of Psychology, University of Gothenburg, Gothenburg, Sweden; b Department of Neurology, Neuromuscular Center, Sahlgrenska University Hospital, Gothenburg, Sweden; c Department of Neuroscience and Rehabilitation, Institute of Neuroscience and Physiology, University of Gothenburg, Gothenburg, Sweden

**Keywords:** Myotonic dystrophy type 1, Neurocognitive disorder, Dementia, Cognition, Montreal cognitive assessment

## Abstract

Cognitive deficits and abnormal cognitive aging have been associated with Myotonic dystrophy type 1 (DM1), but the knowledge of the extent and progression of decline is limited. The aim of this study was to examine the prevalence of signs of neurocognitive disorder (mild cognitive impairment and dementia) in adult patients with DM1. A total of 128 patients with childhood, juvenile, adult, and late onset DM1 underwent a screening using the Montreal Cognitive Assessment (MoCA). Demographic and clinical information was collected. The results revealed that signs of neurocognitive disorder were relatively rare among the participants. However, 23.8 % of patients with late onset DM1 (aged over 60 years) scored below MoCA cut-off (=23), and this group also scored significantly worse compared to patients with adult onset. Age at examination were negatively correlated with MoCA scores, although it only explained a small portion of the variation in test results. Other demographic and clinical factors showed no association with MoCA scores. In conclusion, our findings indicate a low prevalence of signs of neurocognitive disorder in adult patients with DM1, suggesting that cognitive deficits rarely progress to severe disorders over time. However, the performance of patients with late onset DM1 suggests that this phenotype warrants further exploration in future studies, including longitudinal and larger sample analyses.

## 1 Introduction

Myotonic dystrophy type 1 (DM1) is an autosomal dominant inherited progressive multisystemic disorder caused by an unstable CTG trinucleotide repeat expansion in the non-coding region of the DM1 protein kinase (DMPK) gene on chromosome 19 [1]. The disease is the most common form of adult-onset muscular dystrophy with a worldwide prevalence of 9.27/100,000 [2]. Muscular weakness is a characteristic symptom, but DM1 is also associated with ocular, cardiac, endocrine, gastrointestinal, and central nervous system impairments [3]. The disease is categorized into different phenotypes based on age of onset and severity of symptoms, including congenital, childhood, juvenile, adult/classical, and late onset/mild; each exhibiting different clinical features and disease progression [4]. Generally, earlier onset is associated with more severe symptoms.

Brain involvement in DM1 has been investigated using various imaging techniques, revealing an increased prevalence of white matter hyperintense lesions, global and regional brain atrophy, and alterations in the integrity of normal appearing white matter [5,6]. However, the underlying cause of these abnormalities remains unclear. Neurocognitive abnormalities in DM1 phenotypes with an earlier onset have been suggested to be developmental in nature rather than progressive [7] but the knowledge on these variants is limited due to a lack of comprehensive studies [6]. In adult-onset DM1, studies have shown deviations in biomarkers associated with abnormal aging-related processes and dementia [[8], [9], [10]]. Based on these findings, some researchers have described the condition as progeroid, indicating early cognitive aging [[11], [12], [13], [14]] and even dementia [15]. Longitudinal studies have provided further support, demonstrating cognitive decline [[11], [12], [13], [14], [15]] and brain related changes, primarily affecting white matter [16,17], and disease duration has been shown to correlate with neurocognitive dysfunction [12,13,18], suggesting worsening performance over time. However, it remains unclear whether cognitive decline progresses to more severe deficits resembling a neurocognitive disorder [19]. Although more than 30 studies have included assessments of neurocognition in DM1 (see Supplementary data, Table 1), few have explored the prevalence of neurocognitive disorder specifically and systematically, in larger samples of adults. When examining results from the most used screening tool, the Mini-Mental State Examination (MMSE), the prevalence of patients scoring below proposed cut off scores varies highly (0–72 %). Furthermore, sample sizes are generally small to medium (M = 30, range 2–101) with a few exceptions [10,11,21,25,26,28], and comparisons between patients and healthy controls shows highly variable levels of significance and effect sizes (Cohen's d = 0.39–1.62). Consequently, it is challenging to evaluate prevalence figures and perform subgroup analysis. Therefore, the aim of this study was to explore the prevalence of signs of neurocognitive disorder in a representative cohort of patients with DM1 and use these results as an indication of how cognitive impairment may evolve over time. We also explored if cognitive performance was associated with demographic and clinical factors.

**Table 1:** Clinical characteristics on patients with DM1.

	Total group (n = 128)	Childhood (n = 12)	Juvenile (n = 28)	Adult (n = 67)	Late (n = 21)
Age	48 (14)	38.8 (10.7)	39.1 (13.1)	47.6 (11)	66.1 (10.8)
Sex	66 F, 62 M	6 F, 6 M	16 F, 12 M	35 F, 32 M	9 F, 12 M
Duration^a^	20 (11)	31.1 (10.2)	25.8 (12.1)	17.9 (10.1)	12.1 (6.7)
CNS-related					
disorder^b^8 (6 %)	0 (0 %)	1 (8 %)	2 (3 %)	5 (24 %)	
MIRS	3.36 (1)	3.9 (0.7)	3.5 (0.9)	3.5 (0.9)	2.6 (2)
FDSS	8.89 (3.74)	40.5 (11.0)	38 (10.1)	38.1 (11.1)	35 (11.1)
EQ-5D^c^	0.8 (0.9)	0.3 (0.5)	0.6 (0.9)	0.9 (0.9)	0.7 (0.7)

*Note.* DM1 = Myotonic Dystrophy type 1, MIRS = Muscular Impairment Rating Scale, FDSS = Fatigue and Daytime Sleepiness Scale.

a disease duration (years).

b other disorders possibly related to cerebral dysfunction.

c EQ-5D rating on anxiety and depression (for details, see Supplementary data, Table 4 on severity level). Results are presented as mean (sd) when appropriate.

## 2 Materials and methods

### 2.1 Study design

Data was collected during examinations conducted in the initial phase of a prospective study on the long-term consequences of DM1. The Swedish Ethical Review Authority approved the study (number 826-16). It was conducted in accordance with the Declaration of Helsinki (2000) and was fully compliant with the Principles of Good Clinical Practice according to the International Conference on Harmonization. All examinations took place at the Neuromuscular Centre, Sahlgrenska University hospital in Gothenburg, Sweden, between January 1, 2018, and November 30, 2019.

### 2.2 Procedure

Physicians (Drs Eliasdottir, Nordström and Lindberg) administered the Montreal Cognitive Assessment (MoCA), a screening tool for neurocognitive disorder. The procedure also included assessments of muscle strength and function conducted by physicians, physiotherapists, and occupational therapists. Participants completed self-ratings on the presence of daytime sleepiness, fatigue, anxiety, and depression before their visit.

### 2.3 Study subjects

As of December 31, 2017, a total of 246 adult patients (age 18 or older) with genetically confirmed DM1 were on the waiting list at the Neuromuscular Centre. The patients were classified into five phenotypes, according to Guordon and Meola (2017) [4]: congenital (symptoms from birth to 1 year), childhood (symptoms 1–10 years of age), juvenile (symptoms 10–20 years of age), adult/classical (symptoms from 20 to 40 years of age), and late onset/mild (>40 years of age). All 32 patients with congenital DM1 were excluded due to assumed difficulties in participating in the assessment procedure and understanding informed consent, leaving 214. Patients were consecutively invited to participate based on their place on the waiting list, with the aim of including up to three patients per week in 2018 and 2019. Eighty-six patients (49 females, mean age = 50.5, 37 males, mean age = 55.6) were not included (22 % childhood, 11 % juvenile, 24 % adult onset and 43 % with late onset). Fifty patients declined participation, 23 accepted but were unable to participate and 13 were not possible to ask before end of inclusion. Thus, 151 of 214 patients accepted inclusion (71 %) of whom 128 out of 214 (60 %) participated.

### 2.4 Demographic and clinical information

Patients with DM1 underwent an interview regarding their medical health, and additional information was collected from their medical records. For information on demographic and clinical status, see Table 1. Information on age at onset was obtained from medical records. The first self-rated and/or clinically confirmed signs indicating DM1, such as muscle weakness, myotonia, excessive fatigue, neurocognitive impairment or gastrointestinal symptoms were considered as onset of disease.

### 2.5 Instruments

#### 2.5.1 Measurement of neurocognitive disorder

The Montreal Cognitive Assessment (MoCA) was used as a screening tool to detect signs of neurocognitive disorder as defined by the Statistical Manual for psychiatric Disorders 5 (DSM-5) [19]. The instrument has been widely used in aging related cognitive disorders (mild cognitive impairment and dementia) and has demonstrated good psychometric properties, including reliability and validity measures [[31], [32], [33], [34]]. MoCA consists of 16 items and 11 categories to assess multiple cognitive domains, including attention, executive functions, memory, orientation, arithmetic, verbal ability, and visuospatial ability. The maximum total score is 30. The developers of MoCA proposed a cut-off score on 26 to indicate neurocognitive disorder [31], but this cut-off has been criticized for generating inflated rates of false positives results, particularly among older individuals and/or those with lower education levels. Therefore, we followed recommendations of Carson et al. [35], and included a lower cut-off score of 23 to control for false positives. However, results using the higher cut-off are also presented for comparison (see Table 2). Scores below 23 were considered indications of both mild and major neurocognitive disorder. We also compared patients results with a proposed clinical interpretation of the scores [36]. Additionally, results on five cognitive subdomains of the MoCA test and their respective cut-off, were analyzed [37]. See Supplementary data, Table 3, for details on each domain and its related MoCA subtest. The implementation of the test was well tolerated by the patients, with all participants completing the screening and the administrators found the test easy to administer to the patients.

**Table 2:** DM1 patients scores on the MoCA.

DM1 phenotype	Raw score	Cut-off = 23*	Cut-off = 26**
Childhood (n = 12)	26.5 (2.9) [21-30]	2 (16.7 %)	4 (33.3 %)
Juvenile (n = 28)	26.6 (2.4) [21-30]	1 (3.6 %)	11 (39.3 %)
Adult (n = 67)	27.2 (2.4) [18-30]	3 (4.5 %)	11 (16.4 %)
Late (n = 21)	24.1 (5.4) [5-30]	5 (23.8 %)	11 (52.4 %)
Total group (n = 128)	26.5 (3.3) [5-30]	11 (8.6 %)	37 (28.9 %)

*Note.* DM1 = Myotonic Dystrophy type 1, MoCA = Montreal Cognitive Assessment.

Cut-off value based on Carson et al. (2018) *, Nazzredine et al. (2005) **.

Results are presented as mean (sd) [min-max] and number of patients (percent).

**Table 3:** Results on domains of the MoCA test and percentage of patients with DM1 scoring below cut-off (n = 128).

Domain	Raw score	< cut off	Total score [cut-off]^a^
Memory	10.5 (1.6) [1-12]	8 (6.3 %)	12 [8]
Visuospatial	3.4 (0.7) [1-4]	13 (10.2 %)	4 [3]
Language	4.9 (0.3) [4,5]	0 (0 %)	5 [4]
Attention	3.5 (1.0) [0–4]	15 (11.6 %)	4 [3]
Executive	4.0 (1.2) [0–5]	37 (28.9 %)	5 [4]

*Note.* DM1 = Myotonic Dystrophy type 1, MoCA = Montreal Cognitive Assessment.

a Division into different cognitive domains based on MoCA sub scores and cut-off values, see Lam et al. (2013). Results are presented as mean (sd) [min-max] and number of patients (%).

#### 2.5.2 Muscular impairment

The severity of muscular weakness/disease was assessed using the Muscular Impairment Rating Scale (MIRS) [38]. This scale evaluates the severity of muscular impairment according to ﬁve grades: (1) no muscular impairment, (2) minimal signs, (3) distal weakness, (4) mild to moderate proximal weakness, and (5) severe proximal weakness.

#### 2.5.3 Fatigue and daytime sleepiness

The Fatigue and Daytime Sleepiness Scale (FDSS) is a rating scale specifically designed to measure fatigue and daytime sleepiness in patients with DM1 [39]. The questionnaire consists of 12 items rated on a three-point scale (0–2). Raw sum scores vary from 0 to 24. The instrument demonstrates good psychometric properties [40].

#### 2.5.4 Anxiety and depression

Patients provided a simple rating on the presence of anxiety and depression by answering a question from the EQ-5D self-report [41]. The scale ranges from 0 (no anxiety and depression) to 4 (extreme anxiety and depression).

### 2.6 Literature review

We conducted a literature review on studies that have used screening tests for neurocognitive disorder in DM1. One of the authors (SW) conducted searches in Embase (1974–2023), Medline (1946–2023), and PsycInfo (1806–2023) in January 2023. Cross-referencing was also done to identify potentially missed studies. All databases were searched using free text and index terms related to DM1, MMSE, MoCA, dementia and neurocognitive disorder. Only studies involving genetically defined DM1 were included. For studies published before or shortly after discovery of the DMPK gene in 1992, clinical diagnosis of DM1 was acceptable. Information on the screening test used, number of patients/controls, disease phenotype, and reported outcomes were extracted.

### 2.7 Statistics

Data were analyzed using the Statistical Package for the Social Sciences (SPSS) version 25. Frequencies, percentages, and means were used, as appropriate, to describe the characteristics of the participants. The student's t-test, analyses of variance (ANOVA) and post hoc analyses was used to test for differences in continuous variables, with Hedges *g* and Cohen's D, to determine effect size. Correlations between MoCA scores, clinical and demographic factors were analyzed using Pearson correlation coefficient (r) and linear regression (r^2^). The criterion for statistical significance was set at *p* < 0.05.

### 2.8 Data availability

The data supporting the findings of this study are available on reasonable request from the corresponding author (SW). Data are not publicly available due to the privacy of the study participants.

## 3 Results

On the MoCA test, 8.6 % of the patients with DM1 scored below the cut-off for neurocognitive disorder (see Table 2). When analyzing subgroups, 16.7 % with childhood, 3.6 % with juvenile, 4.5 % with adult, and 23.8 % with late onset scored below cut-off. Patients with late onset performed significantly worse than individuals with an earlier, i.e., adult onset (m = 24.1 vs 27.2, *p* < 0.02, *g* = 0.95). When categorizing patient scores according to clinical guidelines (see Supplementary data, Table 2), most patients showed no or mild cognitive impairment, while signs on moderate and severe impairment were very rare.

Patients with DM1 primarily underperformed in one specific domain of the screening: executive functions (see Table 3). Within this domain, patients failed on abstraction ability, such as finding similarities between words (31 %) and generating words beginning with the letter F (31 %). Adult and late onset subgroups were the only ones to show underperformance on the memory domain, with two patients (0.03 %) and six patients (29 %), respectively (see Supplementary data, Fig. 1).

The total score on the MoCA test had a significant negative correlation with age at examination (*p* < 0.05). However, age accounted only for a small portion of the variation in test scores (r^2^ = 0.04). When analyzing subgroups, age was negatively correlated with scores on the MoCA in patients with adult onset DM1 (ρ = −0.307, p < 0.012), and age was significantly correlated with the memory sub-score (ρ = 0.313, p < 0.001). No other correlation was found in the total patient group or in different subgroups when examining scores on the MoCA test and disease duration, sex, muscle function, anxiety and depression, daytime sleepiness, or fatigue. When comparing patients who scored below and above the MoCA cut-off on demographic and disease specific functions as specified above, no significant differences were found. Anxiety and depression were generally rated as mild or nonexistent (see Supplementary data, Table 4).

## 4 Discussion

The present results show that signs of neurocognitive disorder are uncommon in adult patients with DM1. Generally, scores on the MoCA are like those of healthy subjects [42,43] and signs of moderate or severe disorder is very rare. When comparing the prevalence figure with other studies (see Supplementary data, Table 1), the present sample generally shows lower prevalence compared to most, although not all [20,23,24,27,29,30]. These differences may be partly explained by the use of different rating scales and cut-off scores, as well as the inclusion and prevalence of subgroups. In the present sample, the group size was comparably larger, making analyses of the total group and sub-groups more reliable. Furthermore, scores on the MoCA have been shown to be a more valid measure on neurocognitive disorder than MMSE [44], which has been used in most studies on DM1 (see Table 1). This suggests that the present figures may better reflect the prevalence in the population in a more satisfactory way than in previous studies, however excluding congenital DM1. Moreover, the low prevalence figure of neurocognitive disorder is consistent with other studies that have reported that most patients with DM1 are able to perform activities of daily living (ADL) without support, indicating that cognitive dysfunctions do not significantly interfere with ADL [45]. This is generally not the case in patients with neurocognitive disorder [46]. Considering that the examinations were performed in adulthood and few patients score below the MoCA cut-off, these results suggest that for most patients, decline over time does not lead to severe cognitive dysfunctions associated with neurocognitive disorder.

Studies on childhood and juvenile onset phenotypes have shown significant deficits and reduced IQ, when cognition has been measured at a young age [47,48]. Therefore, it is not surprising that a small subgroup of patients also scores below the MoCA cut-off when cognition is measured at adult age. However, signs of neurocognitive disorder were uncommon in the present sample, indicating that cognitive deficits established during childhood have not progressed into severe conditions for most patients (see Table 2, and Supplementary data, Table 2). Regarding adult onset DM1, few patients scored below the cut-off on the MoCA, providing weak support for hypotheses on dementia in this phenotype [15]. The results are more consistent with two recently published studies [49,50] showing heterogenous cognitive and biomarker profiles, with signs of neurocognitive disorder present in only a few patients with adult onset DM1. In the present study, the performance of patients with the late onset was an exception, where nearly one in four scored below the cut off on the MoCA and these patients also scored significantly below patients with adult onset DM1. It somewhat surprising that a subgroup of this size performed below the cut off, considering that this phenotype is mainly associated with other symptoms, such as mild myotonia and cataracts [4,51]. However, it is worth noting that the prevalence of signs of neurocognitive disorder (23.8 %) in this age group (mean age = 66 years) is higher than expected compared to a normative sample (14.5 %) [52]. The difference in MCI scores between adult and late onset DM1 may be due to chance, the limited sample size, and differences in age between groups. Nevertheless, this finding is consistent with results from cross-sectional and longitudinal studies, indicating that patients with late onset DM1 perform worse and show a higher rate of cognitive decline as compared to subgroups with an earlier onset of the disease [15,22,25]. Furthermore, a recent study by Labayru et al. [53] on a small sample of patients with late onset DM1 (n = 8) also showed pronounced white matter integrity loss over time, suggesting neurodegeneration in this phenotype. Other disorders potentially linked to brain dysfunction, such as myocardial infarction and stroke, were found in 24 % of patients with late-onset DM1 (see Table 1). These conditions might affect performance on the MoCA. However, none of the patients with such disorders scored below the MoCA cut-off. In summary, the present results underscore that neurocognitive disorder is a prevalent feature in some patients with late onset DM1. However, few patients show signs of major cognitive impairment, when using clinical cut-offs (see Supplementary data, Table 2).

When analyzing sub scores on the MoCA test, the overall results are consistent with the cognitive profile described in DM1 [54], and deficits were mainly associated with the executive cognitive domain (see Table 3). Low scores on subtests measuring memory were uncommon, and this performance differs from what is typically seen in the most common variant of neurocognitive disorder: Alzheimer's disease (AD). This may indicate that cognitive deficits in DM1 represent other brain alterations than those recognized in AD [55]. However, when analyzing the performance of patients with adult and late onset phenotypes on the MoCA memory domain, scores below the cut off were identified in 2 and 6 patients, respectively (see supplementary data, Fig. 1). This profile was exclusively associated with a later onset of the disease, and may indicate a specific neurocognitive phenotype, as memory dysfunction is generally an uncommon feature in the cognitive performance of patients with DM1 [54].

In this sample, disease duration was not associated with the results on the MoCA test, neither when analyzing the performance of the total patient group nor the subgroups. This indicates that neurocognitive disorder is not more prevalent in patients with a longer duration of the disease. Contradictory results between the present data and other studies [12,13,18] may be explained by differences in sensitivity of the tests used, such as comprehensive neuropsychological test batteries versus a cognitive screening tool like the MoCA. However, correlations between scores on the MoCA and comprehensive neuropsychological batteries have been shown in other disorders [56,57], indicating that the MoCA total score may sufficiently measure cognitive domains included in more comprehensive batteries.

This study has some potential limitations that should be noted. Although the aim was to collect a representative sample, the size of some subgroups was limited, and patients that did not participate from the original cohort, were more prominent in some groups, such as late onset DM1. This means that generalizability is limited regarding some subgroups, and conclusions should be considered preliminary. Collaborative studies between research centers on larger samples are one way to address this problem in the future, as available samples are limited at each site due to the rarity of DM1. Furthermore, decisions on signs of neurocognitive disorder relied on a screening instrument: MoCA. This instrument has been proven to be reliable and valid for detecting neurocognitive disorder, but future studies should include in-depth psychiatric examinations, including interviews in combination with measures of consequences in daily life and ratings of relatives, to further validate the current results. It should also be noted that data was collected at one time point, and future studies should include a longitudinal design to follow disease progression and possible cognitive decline over time.

In conclusion, signs of neurocognitive disorder were uncommon in adult patients with DM1, except for the late onset phenotype, where approximately 1 in 4 scored below the MOCA cut-off. This indicates that assumptions of early cognitive aging processes leading to cognitive dysfunction comparable to neurocognitive disorder, find weak support in the present results. This is important information for healthcare professionals, patients, and relatives in terms of prognosis and long-term planning. However, the prevalence of neurocognitive disorder in late onset DM1 suggest that future studies should explore these patients in larger samples over time.

## Funding

This work was supported by NEURO Sweden. The sponsor was not involved in the conduct or preparation of the study.

## CRediT authorship contribution statement

**Stefan Winblad:** Conceptualization, Data curation, Formal analysis, Investigation, Methodology, Software, Supervision, Validation, Visualization, Writing – original draft, Writing – review & editing. **Olöf Eliasdottir:** Writing – review & editing, Data curation, Investigation, Project administration. **Sara Nordström:** Data curation, Investigation, Writing – review & editing. **Christopher Lindberg:** Investigation, Project administration, Resources, Writing – original draft, Writing – review & editing, Conceptualization, Data curation, Funding acquisition.

## Declaration of competing interest

The authors declare the following financial interests/personal relationships which may be considered as potential competing interestsChristopher Lindberg reports financial support was provided by NEURO Sweden.
