# Supplementary material for: Neurocognitive disorder in Myotonic dystrophy type 1
Source: Heliyon. 2024 May 8;10(10):e30875. doi: 10.1016/j.heliyon.2024.e30875 (PMC11109806; doi:10.1016/j.heliyon.2024.e30875)

**SUPPLEMENTARY MATERIAL**

**Supplementary Table 1**

Characteristics of studies that have used measures on neurocognitive disorder in DM1

Note: † = MMSE, †† = MoCA, ††† = ACER, DM1 = Myotonic dystrophy type 1, HC = Healthy controls, * = significant difference (p < .05), ** = non-significant

**Supplementary Table 2**

Severity level of MoCA scores in DM1 patients according to clinical guidelines*

___________________________________________________________________________

Grading Total group CDM1 JDM1 ADM1 LDM1

N (%) N (%) N (%) N (%) N (%)

_____________________________________________________________________________

No CI (> 25) 93 (72.7) 8 (67) 19 (68) 56 (84) 10 (48)

Mild CI (18-25) 34 (26.5) 4 (33) 9 (32) 11 (16) 10 (48)

Moderate CI (10-17) 0 (0) 0 (0) 0 0 0

Severe CI (< 10) 1 (0.008) 0 (0) 0 0 1 (4)

___________________________________________________________________________

Note: *https://mocacognition.com/faq/, CI = cognitive impairment, CDM1 = Childhood onset DM1, JDM1 = Juvenile onset DM1, ADM1 = Adult onset DM1, LDM1 = Late onset DM1.

**Supplementary Table 3**

Cognitive domain and corresponding MoCA score components*

___________________________________________________

Domain MoCA components

___________________________________________________

Memory Delayed recall

Orientation

Forward digit span

Visuospatial Cube draw

Clock draw

Language Animal picture naming

Sentence repetition

Attention Serial 7´s

Vigilance (“A” tapping test)

Executive Backwards digit span

Trail-making test

Word similarities

F-word list generation

____________________________________________________

*Note.* MoCA = Montreal Cognitive Assessment. * Table modified

from Lam et al., (2013).

**Supplementary Table 4**

Severity level of EQ-5D (anxiety-depression) in patients with DM1 (n = 126)

________________________________________________________________________

Grading Total group CDM1 JDM1 ADM1 LDM1

N (%) N (%) N (%) N (%) N (%)

________________________________________________________________________

No a-d 57 (45) 9 (75) 16 (57) 22 (33) 10 (48)

Mild a-d 50 (39) 3 (25) 9 (32) 30 (45) 8 (38)

Moderate a-d 15 (12) 0 (0) 2 (7) 10 (15) 3 (14)

Severe a-d 4 (3) 0 (0) 0 (0) 4 (6) 0 (0)

Extreme a-d 1 (1) 0 (0) 1 (4) 0 (0) 0 (0)

________________________________________________________________________

a-d = anxiety-depression, CDM1 = Childhood onset DM1, JDM1 = Juvenile onset DM1, ADM1 = Adult onset DM1, LDM1 = Late onset DM1, missing = 2 patients

**Supplementary Figure 1**


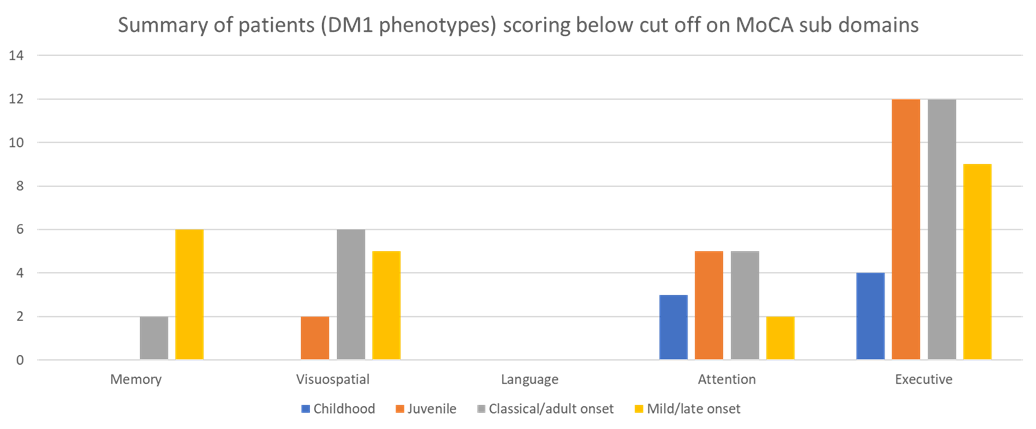

Supplement: Multimedia component 1 [file mmc1.docx]
